# TGBWDriver: A Cancer Driver Gene Identification Method Based on GraphSAGE and Bidirectional Weighted Feature Aggregation

**DOI:** 10.3390/ijms27094135

**Published:** 2026-05-05

**Authors:** Jiaxin Chen, Yingzan Ren, Haihui Wang, Cong Zhan, Yusen Zhang

**Affiliations:** School of Mathematics and Statistics, Shandong University, Weihai 264209, China; chenjiaxin101512@163.com (J.C.); yingzanren@163.com (Y.R.); 15169620300@163.com (H.W.); 19370417270@163.com (C.Z.)

**Keywords:** cancer driver genes, GraphSAGE, PPI network, multi-omics data

## Abstract

Identifying cancer driver genes is fundamental for understanding tumor initiation and guiding therapeutic strategies. However, most existing methods assess gene importance from a global or static perspective, overlooking sample-specific functional differences in the same gene. To address this, we propose TGBWDriver, which integrates a two-layer GraphSAGE with bidirectional weighted feature aggregation to capture structural characteristics while distinguishing context-dependent gene functions. An exponential pairwise voting strategy prioritizes candidate driver genes, improving ranking stability and accuracy. Systematic experiments on BRCA, LUAD, and PRAD datasets show that TGBWDriver outperforms five existing methods in precision, recall, and F1-score. Ablation studies confirm the critical role of each component. Moreover, TGBWDriver demonstrates strong capability in identifying potential novel cancer driver genes, with predictions showing significant biological relevance in GO enrichment and KEGG pathway analyses. The method provides an effective computational framework for cancer driver gene identification. The source code and datasets are freely available at https://github.com/SCSMDyeah/TGBW [Accessed on 4 May 2026].

## 1. Introduction

Cancer is a prototypical evolutionary disease whose initiation and progression are primarily driven by the gradual accumulation of somatic genetic alterations, including single-nucleotide variants and copy number variations. Among the large number of somatic mutations present in cancer genomes, only a small subset, known as driver mutations, confer selective growth advantages to cells and thereby promote clonal tumor evolution, whereas the vast majority are passenger mutations that do not directly contribute to tumorigenesis [1,2,3]. Accurately identifying driver genes that harbor driver mutations is not only central to understanding the molecular mechanisms underlying cancer initiation and progression, but also crucial for achieving precise diagnosis, subtype classification, and targeted therapy, making it one of the fundamental tasks in cancer genomics.

Among existing approaches for cancer driver gene identification, mutation frequency-based methods and network-based methods represent the two most representative technical paradigms. Mutation frequency-based methods, exemplified by MutSigCV [4], MuSiC [5], and OncodriveFM [6], identify driver genes by comparing observed mutational burden against expected background rates. While effective for high-frequency drivers, they struggle to detect low- and moderate-frequency mutations and are sensitive to mutational heterogeneity across cancer types. In contrast, network-based methods map mutation or expression data onto protein–protein interaction networks to identify critical genes or modules through diffusion, subnetwork search, or path-based scoring. Representative examples include HotNet2 [7], which identifies significantly mutated subnetworks in protein interaction networks, as well as DawnRank [8] and PRODIGY [9], which perform personalized driver gene prioritization by integrating gene interaction networks with single-sample mutation and differential expression data. In addition, DriverNet [10] identifies functional driver genes by characterizing the network coverage of mutation-induced downstream expression perturbations, while Subdyquency [11] and DriverRWH [12] prioritize candidate driver genes based on mutated subnetwork enrichment and random walk-based diffusion strategies, respectively. Although network-based methods enable a systems-level integration of gene interactions and are well suited for uncovering candidate drivers located in key pathways or functional modules, their performance is largely constrained by the completeness and noise of prior network and pathway annotations. Moreover, substantial differences exist among these methods in terms of their ability to exploit individual-level information and the depth at which multi-omics data are integrated.

Recently, machine learning (ML) techniques, particularly deep learning-based methods, have achieved substantial progress in cancer driver gene identification. Among traditional ML approaches, 20/20+ [13], DriverML [14], and DORGE [15] are representative methods, which formulate driver gene identification as a supervised learning problem using multi-source features (e.g., mutation frequency, gene expression) and ensemble learning or regularized regression to distinguish driver genes from passenger genes. Overall, these ML-based methods outperform traditional statistical and single-network approaches in global driver gene prediction.

However, they generally treat genes as independent samples, while network topology information from protein interaction networks and regulatory networks is often incorporated only as auxiliary features or prior knowledge. In addition, most of these methods rely on cohort-level driver gene labels as supervision signals and produce global driver gene scores, which limits their ability to capture molecular heterogeneity arising from distinct mutation combinations and expression contexts across individual patients. To more effectively exploit interaction information among genes, a growing number of graph neural network (GNN)-based methods have been proposed in recent years, explicitly incorporating network topology into ML frameworks to model the propagation and aggregation of mutational signals. GNNs are a class of network representation learning approaches that jointly learn low-dimensional node embeddings by integrating network structure and node features [16]. For example, EMOGI [17] is a GCN-based driver gene identification method that integrates genomic, epigenomic, and transcriptomic data as node features and leverages protein interaction networks to capture interactions among genes, achieving promising performance in multi-cancer driver gene prediction. MTGCN [18] proposes a GCN-based multi-task learning framework that optimizes node embeddings by jointly learning node classification and edge link prediction tasks. IMCDriver [19] maps multi-omics features, such as mutation and expression data, onto protein interaction networks and applies graph neural networks to learn high-dimensional gene representations, enabling end-to-end driver gene prediction. Furthermore, NIGCNDriver [20] combines normalized interaction networks with GCNs to integrate multi-source information under structural constraints, thereby improving the robustness and accuracy of driver gene identification. We have provided a systematic methodological comparison of existing driver gene identification methods—categorized into mutation frequency–based, network-based, machine learning–based, and GNN-based approaches—in Supplementary Table S1.

Although the aforementioned methods have improved the accuracy of driver gene identification to varying degrees, most of them assume that the interactions between genes and samples are static and unidirectional, making it difficult to learn fine-grained weights at the level of gene–sample pairs, and failing to distinguish directional information flow from genes to samples and from samples to genes. Meanwhile, although GCNs have been successfully applied to address a variety of association prediction tasks in bioinformatics [21,22,23,24], their application to large-scale gene networks is hindered by high computational and memory costs, leading to prohibitively long training times and limited scalability.

To address the aforementioned limitations, we propose a novel cancer driver gene identification method, termed TGBWDriver. Compared with existing approaches for identifying cancer driver genes, TGBWDriver offers the following major advantages.

1. By introducing a two-layer GraphSAGE architecture, the model enables hierarchical information fusion and interaction modeling: the first layer captures direct feature interactions between genes and samples, while the second layer further integrates the already interacted representations, thereby enhancing the representational capacity across different node types.
2. We propose a Bidirectional Weighted Feature Aggregation (BWFA) mechanism to dynamically adjust the mutual influence between gene nodes and sample nodes. In this mechanism, gene–sample relationships are not treated as fixed but are adaptively weighted according to sample-specific contextual information, allowing the model to accurately capture asymmetric and dynamic dependency patterns between genes and samples. This design enables the framework to better accommodate heterogeneity across different cancer types and improves the accuracy of driver gene identification.
3. An Exponential Pairwise Voting (EPV) strategy is introduced to optimize the prioritization of candidate genes during the ranking stage. By performing pairwise comparisons of gene rankings at the sample level and employing an exponential mechanism to modulate the contribution of ranking positions to the overall score, EPV allows the model to more accurately identify genes with strong driver effects across different samples, thereby further enhancing predictive performance.

We applied TGBWDriver to three cancer datasets from The Cancer Genome Atlas (TCGA) [25] and evaluated the results using cancer driver genes curated in the Network of Cancer Genes (NCG) [26] as benchmarks. Comparative experiments demonstrate that TGBWDriver outperforms existing methods in detecting cancer driver genes. Moreover, TGBWDriver is capable of not only accurately identifying known driver genes but also discovering potential novel cancer-related driver genes. Overall, TGBWDriver serves as an effective and practical complement to existing cancer driver gene identification approaches.

## 2. Results

### 2.1. Cross-Cancer Benchmarking of Competing Methods

**Figure 1:**
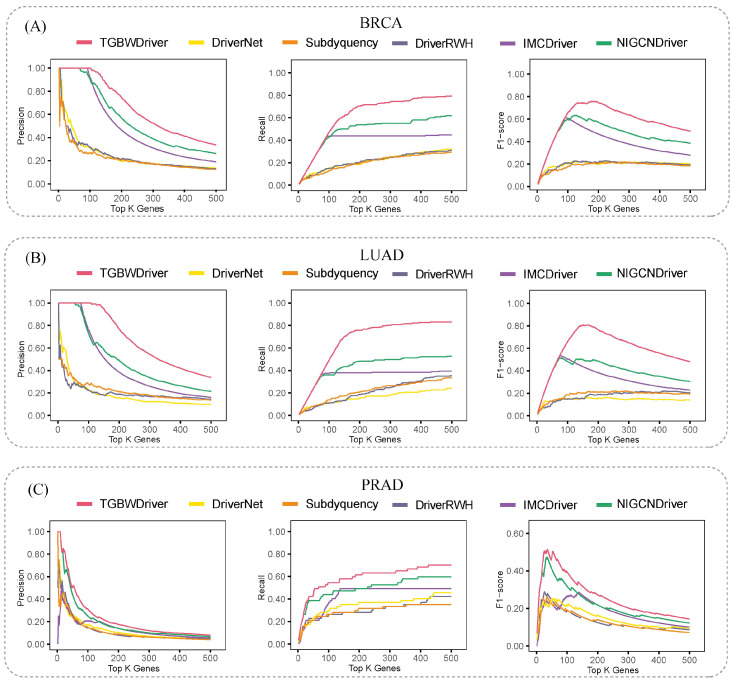
Precision, recall, and F1-score of different methods on BRCA, LUAD, and PRAD across Top-K thresholds.

To evaluate the effectiveness of the proposed TGBWDriver method, we compared it with five advanced methods, including NIGCNdriver, IMCdriver, DriverNet, Subdyquency, and DriverRWH. For performance evaluation, each dataset was randomly partitioned into five mutually exclusive subsets. In each iteration, four subsets (80% of the samples) were used for training, while the remaining subset (20% of the samples) was used for testing. This procedure was repeated five times so that each sample was used for testing exactly once. The performance of different methods was systematically compared on three cancer types using precision, recall, and F1-score as evaluation metrics. Known driver genes curated in NCG 6.0 for each cancer type were treated as ground truth, and precision, recall, and F1-score were calculated based on the top-N ranked genes predicted by each method. As shown in Figure 1, across the BRCA, LUAD, and PRAD datasets, TGBWDriver (indicated by the red curves) consistently outperforms the competing methods under different Top-K gene thresholds. Specifically, TGBWDriver maintains higher precision and recall across all three cancer types and achieves superior F1-scores over a wide range of Top-K values, indicating a more balanced performance between accuracy and coverage in driver gene identification. The advantage of TGBWDriver is particularly pronounced in the BRCA and LUAD datasets, where its F1-score reaches a peak at intermediate Top-K ranges and is substantially higher than that of other methods. In the PRAD dataset, although driver gene identification is generally more challenging, TGBWDriver still maintains leading performance in terms of precision and overall effectiveness, demonstrating its stability across different cancer types.

### 2.2. Cross-Network Robustness Evaluation of TGBWDriver

#### 2.2.1. Prediction Accuracy on Individual Networks Validated by NCG Database

**Figure 2:**
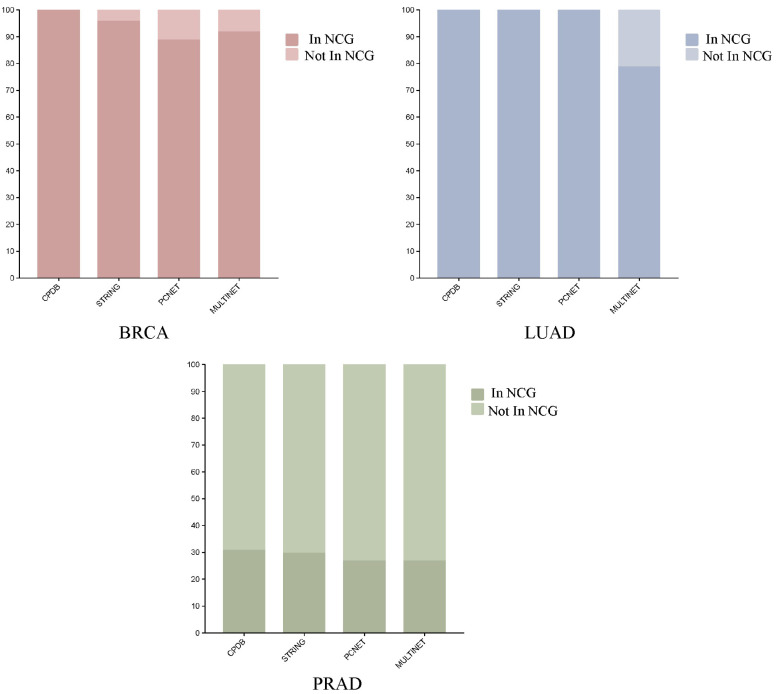
Performance comparison of TGBWDriver across different PPI networks.

To investigate the effectiveness of TGBWDriver in identifying novel cancer driver genes under different biological network settings, we further applied TGBWDriver to three additional well-established networks—STRING, PCNet, and MULTINET—in addition to CPDB. For each network, the top 100 predicted genes were selected for analysis. By comparing the prediction results with the known driver genes curated in the NCG database (as shown in Figure 2), we found that in both the BRCA and LUAD datasets, at least 80% of the predicted genes were confirmed as known driver genes. Although the number of known driver genes covered by the predictions in the PRAD dataset was relatively smaller, this is mainly due to the limited number of currently known PRAD driver genes, which totals only 57, thereby constraining the maximum number of identifiable known drivers. These results demonstrate that TGBWDriver is able to accurately predict a substantial proportion of cancer driver genes across different biological networks.

#### 2.2.2. Prediction Consistency Across Four Biological Networks Based on Gene Overlap Analysis

**Figure 3:**
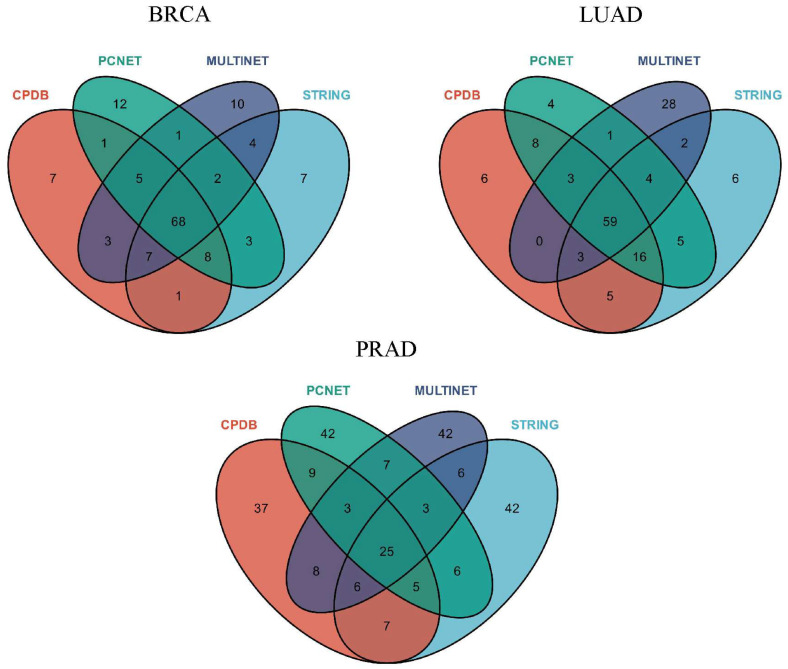
Venn diagram of TGBWDriver prediction results across different biological networks.

To investigate the impact of different biological networks on the predictions of TGBWDriver, we used a Venn diagram to illustrate the overlap among the top 100 driver genes predicted by TGBWDriver in each of the four networks. As shown in Figure 3, taking BRCA as an example, the intersection of the top 100 genes predicted by the four networks contains 68 genes. Notably, comparison with the NCG driver gene database shows that all 68 overlapping genes are known driver genes (e.g., TP53, PIK3CA, GATA3). Similarly, in LUAD, the overlap among the top 100 genes predicted by the four networks includes 59 genes, all of which are known driver genes. In PRAD, the number of overlapping genes is 25, of which 23 are known driver genes, while only 2 genes have not yet been recognized as known driver genes. Collectively, these results indicate that the proposed method yields highly consistent prediction results across different biological networks.

To further exclude the influence of network structure or inherent bias in node labels on prediction performance, we designed two types of negative control experiments on the CPDB network: Edge Shuffle and Node Shuffle. For the Edge Shuffle, we randomly rewired the edges while preserving the degree of each node, generating three randomized networks. For the Node Shuffle, we kept the network structure intact but randomly permuted the gene labels, also generating three randomized versions. We then applied TGBWDriver to all six negative control networks and evaluated its performance on the BRCA, LUAD, and PRAD datasets. The results (see Supplementary Figures S1–S3) show that the prediction performance of all negative control networks was significantly lower than that of the original CPDB network, with a substantial decrease in F1-score, and the effect was particularly pronounced for Node Shuffle. These results confirm that the superior performance of TGBWDriver genuinely depends on real gene–gene interaction relationships and proper gene functional annotations, rather than on statistical properties of network topology or random noise in the input data.

### 2.3. Ablation Experiments

TGBWDriver improves prediction performance mainly through three key components: (1) A two-layer GraphSAGE architecture is employed to achieve layer-wise information fusion and interaction modeling. (2) On top of the two-layer GraphSAGE framework, a bidirectional weighted feature aggregation mechanism is incorporated to address the limitations of conventional graph convolution approaches, in which fixed and homogeneous neighbor aggregation fails to capture the dynamic and gene-specific associations between genes and samples. (3) During the candidate driver gene ranking stage, an Exponential Pairwise Voting (EPV) strategy is introduced to more finely integrate sample-specific gene ranking information. To systematically evaluate the contribution of each component to the overall model performance, we designed a three-factor ablation study on the BRCA, LUAD, and PRAD datasets, in which we varied: the number of GraphSAGE layers (one layer vs. two layers), whether the bidirectional weighted feature aggregation mechanism was enabled, and the final gene ranking strategy (standard Condorcet ranking [27] vs. EPV). As shown in Table 1, among all eight experimental configurations, the combination of “two-layer GraphSAGE + bidirectional weighted feature aggregation + EPV ranking” achieves stable and significant performance advantages across three cancer types, attaining the best performance of 0.469, 0.481, and 0.144 on the BRCA, LUAD, and PRAD datasets, respectively. Removing any individual module leads to varying degrees of performance degradation: when EPV is removed, the performance drops to 0.275, 0.330, and 0.083; when BWFA is removed, it drops to 0.383, 0.421, and 0.097; and when the two-layer GraphSAGE is reduced to a single layer, the performance decreases to 0.463, 0.472, and 0.134. These results demonstrate that the two-layer graph structure, the bidirectional weighted feature aggregation mechanism, and the exponential pairwise voting method each contribute positively to model performance, and it is the synergistic effect of all three modules that achieves the optimal prediction results.

### 2.4. Parameter Analysis

**Figure 4:**
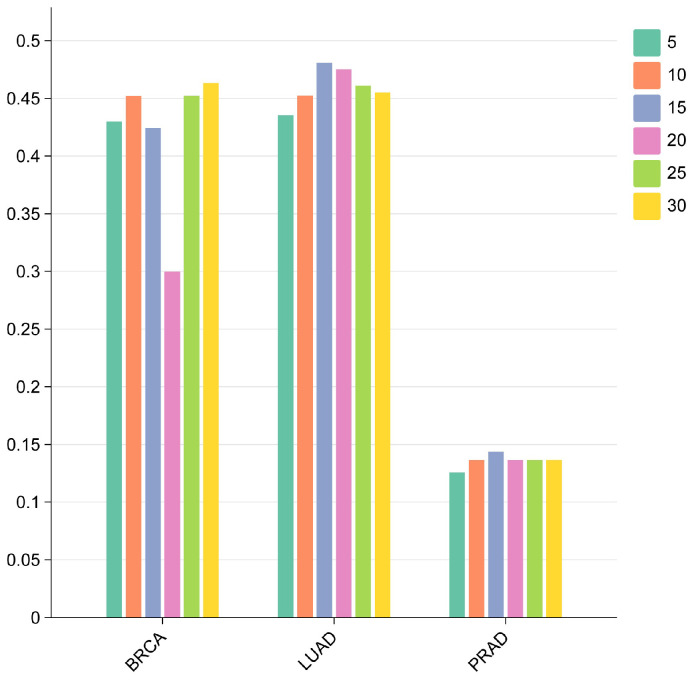
Effect of different GraphSAGE neighbor sampling sizes on the performance of TGBWDriver.

In this study, we adopt a two-layer GraphSAGE architecture to learn node representations. The number of neighbors sampled at each layer serves as a key hyperparameter, determining the scale of neighbors aggregated during each step of message passing and thereby influencing the model’s coverage of local network structural information and its representation capacity. To determine the optimal setting of this parameter, we conducted comparative experiments on gene networks corresponding to three cancer types (BRCA, LUAD, and PRAD). For each cancer type, the neighbor sampling size was set to six levels (5, 10, 15, 20, 25, and 30), and the F1-score of driver gene classification was adopted as the primary evaluation metric. The results indicate that the optimal number of neighbors that yields the highest predictive F1-score varies across cancer types. As illustrated in Figure 4, the BRCA dataset achieved the highest F1-score when the sampling size was set to 30, whereas LUAD and PRAD both exhibited optimal performance at a sampling size of 15. These findings indicate that heterogeneity in network structures necessitates cancer-specific parameter configurations. Based on these results, we selected the optimal neighbor sampling size for each dataset in the final model training and evaluation.

In addition, to enable a fair and comprehensive evaluation of different hyperparameter configurations within each cancer type, we constructed cancer-specific evaluation schemes for BRCA, LUAD, and PRAD by independently optimizing the weight parameter in the Exponential Pairwise Voting (EPV) strategy. This weight parameter is used to adjust the relative importance of multiple evaluation criteria in the integrated scoring process, thereby reflecting differences in evaluation priorities across cancer types in driver gene identification tasks. As shown in Table 2, through systematic experiments over 11 candidate weight values ranging from 0 to 1, we identified the optimal weight settings for each cancer type (BRCA: α = 0.9, LUAD: α = 0.3, and PRAD: α = 0.6). These optimized, cancer-specific weights were then used to perform unified comparisons across different hyperparameter configurations, ensuring internal consistency in the model selection process and the reliability of the evaluation results.

### 2.5. Analysis of Novel Cancer Genes

To evaluate the ability of TGBWDriver to predict potential novel driver genes, we analyzed the top 500 ranked genes not annotated in the NCG database across three cancer datasets. We have listed the top 500 predicted genes for each of the three cancer types (BRCA, LUAD, and PRAD) using the CPDB network in Supplementary Tables S2–S4, respectively. Taking BRCA as an example, we found that multiple genes with high prediction scores but not listed in the NCG database have independent studies supporting their critical roles in breast cancer. Specifically, these genes involve multiple dimensions of regulatory mechanisms underlying breast cancer development and progression. In terms of hormone signaling pathways, NRIP1 [28] encodes a protein involved in the transcriptional activation of steroid receptors, including the estrogen receptor, which plays an important role in normal fallopian tube development, and NRIP1 mutations have been associated with breast cancer in genome-wide association studies. At the level of transcriptional regulation, CNOT1 [29] (CCR4-NOT transcription complex subunit 1) has been identified as a correlated gene in RNA-seq and microarray transcriptomic data of BRCA. Regarding the regulation of malignant cellular behaviors, SUPT5H [30] has been demonstrated to play an essential role in breast cancer tumorigenicity by regulating the expression levels of genes controlling proliferation, migration, cell cycle, and apoptosis in breast cancer MDA-MB-231 cells; SYCP2 [31] plays a vital role in the pathogenesis and progression of human breast carcinoma, thus serving as a promising prognostic molecular marker of poor survival. In terms of therapeutic targets, ubiquitin-specific protease 1 (USP1) [32] represents an emerging therapeutic target for BRCA-deficient malignancies, and compound 43 shows promising preclinical translational potential for BRCA-mutated breast cancer. Furthermore, NOTCH2 and NOTCH4 [33] are well-known genes in BRCA and other cancers, with most mutations in NOTCH2 and NOTCH4 being missense mutations distributed across different domains, including the EGF-like domain, calcium-binding EGF domain, and NOTCH protein domains. Together, the above literature evidence supports, from multiple levels including hormone regulation, transcriptional regulation, malignant cellular behaviors, and therapeutic targets, that these high-scoring genes predicted by TGBWDriver possess clear biological functions and clinical relevance, demonstrating that this method can effectively identify novel driver genes not yet cataloged in existing databases. For similar literature validation results for the LUAD [34,35,36,37,38,39] and PRAD [40,41,42,43,44,45] datasets, see the Supplementary Materials.

**Figure 5:**
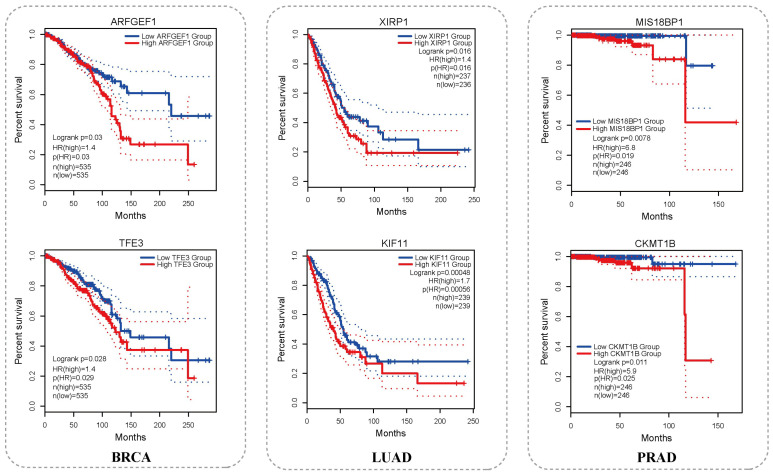
Survival analysis of candidate driver genes based on GEPIA2. OS analysis was performed using GEPIA2 for top-500 predicted genes not in NCG across BRCA, LUAD, and PRAD. The figure shows six genes (two per cancer type) whose expression levels are significantly associated with patient survival.

Gene Expression Profiling Interactive Analysis 2 (GEPIA2) [46] is an updated version of GEPIA for the analysis of RNA sequencing data. In this study, we used GEPIA2 to perform survival analysis on genes ranked within the top 500 predictions but not included in the NCG database across the three cancer datasets. As shown in Figure 5, the survival analysis results indicate that, in BRCA, LUAD, and PRAD, two representative genes were selected in each cancer type, whose expression levels are significantly associated with overall survival (OS) of patients. Specifically, lower expression levels of these genes were associated with significantly reduced overall survival, with hazard ratios (HRs) greater than 1, suggesting that their low expression may serve as an adverse prognostic factor in cancer (log-rank p < 0.05, HR > 1, median cutoff).

### 2.6. GO and KEGG Pathway Enrichment Analysis

**Figure 6:**
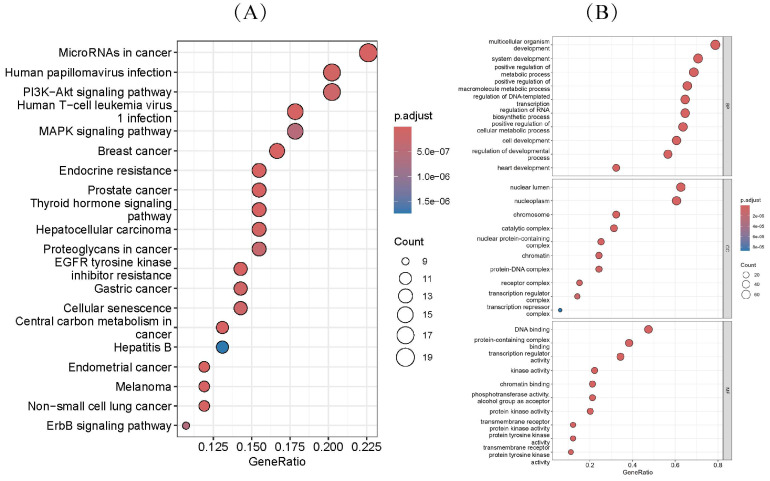
GO and KEGG enrichment analyses of genes predicted by TGBWDriver in BRCA. (**A**) KEGG pathway enrichment results. (**B**) GO enrichment results for Biological Process (BP), Cellular Component (CC), and Molecular Function (MF), showing the top 10 most significant GO terms in each category. The size of each bubble represents the number of enriched genes (Count), and the color indicates the adjusted significance level (adjusted *p* value).

To further investigate the biological functions of genes predicted by TGBWDriver, we performed Gene Ontology (GO) [47] and Kyoto Encyclopedia of Genes and Genomes (KEGG) [48] pathway enrichment analyses on the top 100 predicted genes in each of the three cancer datasets using the R package clusterProfiler (v4.10.0) [49]. We found that the BRCA driver genes identified by TGBWDriver were significantly enriched in 107 KEGG pathways (p < 0.05, q < 0.05) (Supplementary File S3). Among these, the top 20 most significantly enriched pathways were all known or potentially cancer-related pathways (Figure 6A). For example, the proteoglycans in cancer pathway (adjusted p = 2.77 × 10^−7^), PI3K-Akt signaling pathway (adjusted p = 1.62 × 10^−7^), melanoma pathway (adjusted p = $1.37\times 10^{-8}$), and microRNAs in cancer pathway (adjusted p = $1.13\times 10^{-9}$) are all well-recognized cancer-associated pathways. More importantly, the predicted genes were significantly enriched in BRCA-specific pathways: the KEGG Breast cancer pathway (hsa05224) (adjusted p = $1.13\times 10^{-9}$) and the Estrogen signaling pathway (adjusted p = $8.85\times 10^{-4}$). The Breast cancer pathway is classified by KEGG [48] as “Cancer: specific types,” representing a breast cancer-specific disease pathway, and both BRCA1 and BRCA2 are directly annotated to this pathway. The estrogen receptor (ER) signaling pathway [50] is a critical regulator of cell proliferation, differentiation, and survival in breast cancer (BC) and other hormone-sensitive cancers. These results indicate that the genes predicted by TGBWDriver can specifically capture the core driving mechanism of the hormone receptor signaling axis in breast cancer.

GO enrichment analysis was conducted across three categories: Biological Process (BP), Molecular Function (MF), and Cellular Component (CC). Figure 6B presents the top 10 significantly enriched GO terms in each of these three GO categories for the BRCA dataset. In terms of biological processes, the predicted genes were mainly involved in multicellular organism development, system development, and positive regulation of metabolic processes. With respect to cellular components, these genes were significantly enriched in the nuclear lumen, nucleoplasm, and chromatin-related regions. In the molecular function category, the genes exhibited key functional roles, including DNA binding, protein-containing complex binding, and transcription regulator activity.

Similarly, the top 100 predicted genes for LUAD and PRAD were also significantly enriched in cancer-related pathways, as detailed in Supplementary Figures S4 and S5, respectively. Based on the results of GO enrichment analysis and KEGG pathway analysis, it can be concluded that TGBWDriver is capable of effectively predicting tumor-related genes. These genes are not only directly involved in tumor initiation and progression, but also play important roles in cancer development through their functions in biological processes, molecular functions, and cellular components.

## 3. Discussion

In this study, we propose an innovative and effective method for cancer driver gene prediction, termed TGBWDriver. By incorporating a two-layer GraphSAGE model, the method achieves layer-wise information fusion and interaction, effectively enhancing the mutual information between genes and samples. In addition, the proposed bidirectional weighted feature aggregation mechanism addresses the limitation of conventional graph-based models that rely on fixed and homogeneous neighbor aggregation, which is insufficient for capturing dynamic and sample-specific gene–sample associations. Through a weighting strategy, this mechanism enables the learned gene and sample embeddings to more accurately reflect their contextual relevance, further improving the representational capacity of the model. At the driver gene ranking stage, we adopt the Exponential Pairwise Voting (EPV) strategy to prioritize candidate driver genes. Extensive experiments on three cancer datasets—BRCA, LUAD, and PRAD—demonstrate that TGBWDriver consistently achieves stable and superior performance across multiple evaluation metrics and experimental settings. In comparative experiments with five representative methods (NIGCNdriver, IMCdriver, DriverNet, Subdyquency, and DriverRWH), TGBWDriver overall outperformed the competing methods in terms of key metrics, including accuracy, recall, and F1-score. Furthermore, experiments conducted on multiple protein–protein interaction networks (CPDB, STRING, PCNet, and MULTINET) further validate the cross-network robustness of TGBWDriver. A highly consistent core set of predicted genes is observed across different networks, and this consensus set is significantly enriched with known cancer driver genes, demonstrating that TGBWDriver can reliably capture key driver signals embedded in network structures and that its predictions exhibit high biological credibility. It should be noted that the negative control experiments further support the above conclusions. By performing Edge Shuffle and Node Shuffle on the CPDB network, we either destroyed the real gene–gene interaction relationships or disrupted the correspondence between gene features and their network positions. The results showed that across all three cancer types, the predictive performance of TGBWDriver on all negative control networks was significantly lower than that on the original network. This indicates that the performance advantage of TGBWDriver does not arise from statistical properties of network topology (e.g., degree distribution) or random noise in the input data, but genuinely depends on the biological adjacency relationships encoded in the PPI network. In other words, the key to TGBWDriver’s ability to effectively identify cancer driver genes lies in its capacity to extract functionally meaningful topological features from real gene–gene interaction networks, rather than merely exploiting the mathematical properties of the network structure. The ablation study systematically reveals the contribution of each model component to performance improvement. Among these components, the bidirectional weighted feature aggregation mechanism plays a critical role across all cancer datasets and constitutes the primary contributor to the performance improvement of the model. The two-layer GraphSAGE architecture further enhances gene representation learning, while the exponential pairwise voting strategy effectively integrates patient-specific ranking information, thereby improving the consistency and robustness of the final prediction results. Parameter sensitivity analysis indicates that different cancer types exhibit distinct requirements for model parameters, and adopting cancer-specific parameter configurations can substantially enhance predictive performance. In terms of novel driver gene discovery, TGBWDriver successfully identifies candidate genes that are not included in the NCG database but have been supported by existing literature as being closely associated with cancer initiation and progression. Further survival analysis using GEPIA2 shows that the expression levels of some of these genes are significantly associated with overall patient survival, suggesting their potential value in tumor progression and prognostic assessment. GO and KEGG enrichment analyses further demonstrate that genes predicted by TGBWDriver are significantly enriched in multiple well-known cancer-related biological processes and signaling pathways, including the PI3K-Akt pathway and the Estrogen signaling pathway. Although some of these pathways are relatively broad and have been reported in multiple cancer types, their identification in this study serves an important validation purpose: confirming that the genes prioritized by TGBWDriver indeed possess established cancer relevance. More importantly, beyond these general pathways, TGBWDriver also shows significant enrichment in cancer-type-specific pathways—for example, the Breast cancer pathway (hsa05224) in BRCA and the Estrogen signaling pathway in hormone-sensitive breast cancer. These results provide functional evidence that TGBWDriver captures not only general cancer hallmarks but also cancer-type-specific driver mechanisms.

However, the present study only utilizes gene expression matrices and somatic mutation matrices. Future work may further improve the model by integrating additional types of multi-omics data, such as DNA methylation data, epigenetic information, and clinical features.

## 4. Materials and Methods

### 4.1. Data Collection and Preprocessing

The datasets used in TGBWDriver consist of somatic mutation data and gene expression data collected from The Cancer Genome Atlas (TCGA), covering three different cancer types: breast cancer (BRCA), lung adenocarcinoma (LUAD), and prostate adenocarcinoma (PRAD). Specifically, the datasets include 957 BRCA samples, 499 LUAD samples and 450 PRAD samples. To evaluate the proposed model, cancer type-specific driver gene sets were constructed based on the Network of Cancer Genes (NCG 6.0) database. These reference sets contain 221 known BRCA driver genes, 203 known LUAD driver genes and 57 known PRAD driver genes, respectively.

To comprehensively assess the performance of TGBWDriver, we conducted experiments on four widely used protein interaction networks, including CPDB [51], STRING [52], PCNET [53] and MULTINET [54]. Among them, the CPDB network was used as the primary experimental platform to systematically validate the effectiveness of the proposed method, while the remaining networks were employed for comparative analyses to further examine the robustness and generalization ability of the model across different protein interaction network settings. For the CPDB network, interactions with confidence scores lower than 0.5 were filtered out. For the STRING database, only interactions with confidence scores greater than 0.85 were retained. The MULTINET network was collected from the HotNet2 GitHub repository, and the PCNET network was used without additional preprocessing. Table 3 summarizes the number of genes and interactions in each of the four networks.

To enhance the efficiency of model training, we performed preprocessing on each original dataset from The Cancer Genome Atlas (TCGA). Our focus was on predicting genes that undergo mutations in at least one sample. Based on previous studies indicating that only mutations significantly affecting downstream gene expression are more likely to be driver genes, we excluded mutation genes that do not have a significant impact on downstream gene expression, as they lack direct connections to aberrantly expressed genes within the protein–protein interaction (PPI) network [10]. Aberrantly expressed genes refer to those whose expression is significantly affected, identified by calculating the z-score of gene expression in samples. Genes with a z-score greater than or equal to 2 were considered aberrant genes. Here, x represents the raw gene expression value of a sample, while µ and σ denote the mean and standard deviation of gene expression values across samples, respectively. Through this approach, we were able to more precisely identify cancer driver genes.

(1) $$Z‐\mathrm{score}=\frac{(x-\mu )}{\sigma }$$

After the aforementioned processing, we obtained three matrices. The gene expression matrix is referred to as the sample feature matrix $X_{s}\in R^{N\times G}$, where G denotes the number of genes and N denotes the number of samples. The processed mutation matrix is defined as the gene feature matrix $X_{g}\in R^{G\times N}$, with G and N having the same meanings as above. In the gene feature matrix, if gene i undergoes a mutation in sample j, it is recorded as 1; otherwise, it is recorded as 0. In addition, we constructed a driver gene–sample association matrix $A\in R^{G\times N}$ with the same dimensions as the mutation matrix. In matrix A, if gene i undergoes a mutation in sample j and gene i is a known driver gene, it is recorded as 1; otherwise, it is recorded as 0.

### 4.2. Framework of TGBWDriver

**Figure 7:**
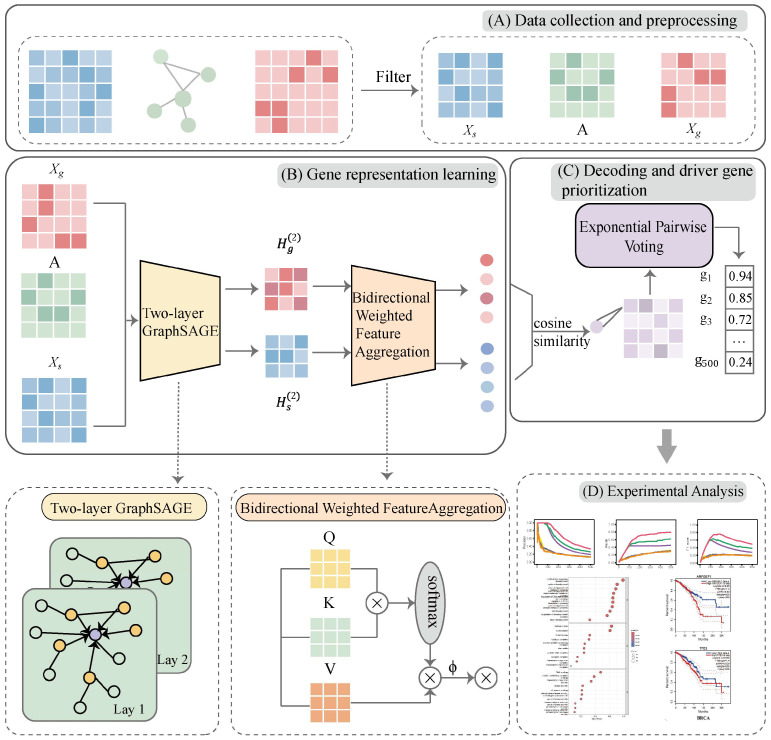
Overall workflow of TGBWDriver. (**A**) Data collection and preprocessing: Gene feature matrices, sample feature matrices, and gene–sample association matrices are constructed based on the protein interaction network, somatic mutation data, and gene expression data. (**B**) Gene representation learning: A two-layer GraphSAGE architecture is applied to both gene and sample nodes, and the final node representations are learned through a bidirectional weighted feature aggregation mechanism. (**C**) Decoding and driver gene prioritization: The final representations of genes and samples are decoded using cosine similarity to generate a gene–sample association probability matrix, and candidate driver genes are subsequently prioritized using an exponential pairwise voting strategy. (**D**) An example illustrating the driver gene prediction performance of the proposed model on the BRCA dataset.

TGBWDriver is a comprehensive deep learning framework designed to predict cancer driver genes by integrating diverse biological data sources. As illustrated in Figure 7, the architecture consists of three main stages: data collection and preprocessing, gene representation learning, and decoding with driver gene ranking. (A) Data Collection and Preprocessing: Based on the PPI network, somatic mutation matrix, and gene expression matrix, the raw data are first preprocessed. Specifically, the Z-Score method is used to identify abnormally expressed genes (Z-Score ≥ 2), and mutated genes that do not significantly affect downstream gene expression are excluded. After the above processing, three input matrices are constructed: a gene feature matrix $X_{g}\in R^{G\times N}$, a sample feature matrix $X_{s}\in R^{N\times G}$, and a driver gene–sample association matrix $A\in \;R^{G\times N}$, where G is the number of genes and N is the number of samples. (B) Gene Representation Learning: A two-layer GraphSAGE network is introduced on both gene and sample nodes to learn initial node embeddings by aggregating information from neighboring nodes. On this basis, a Bidirectional Weighted Feature Aggregation (BWFA) mechanism is proposed for the first time. This mechanism computes association scores in two directions (gene-to-sample and sample-to-gene) to dynamically adjust the mutual influence between nodes, achieving a weighted fusion of gene and sample embeddings. Consequently, it simultaneously updates the node representations in both directions, obtaining more discriminative gene embeddings and sample embeddings. (C) Decoding and Driver Gene Ranking: The learned final gene representations and final sample representations are passed through a cosine similarity decoder to compute similarity scores, yielding a gene–sample association probability matrix, where each element represents the predicted probability that the corresponding gene is a driver gene in that sample. To address the extreme imbalance between positive and negative samples, a weighted binary cross-entropy loss function is adopted. A hyperparameter $w_{\mathrm{ij}}$ is used to amplify the loss contribution of positive samples, forcing the model to prioritize the minority class. Finally, an Exponential Pairwise Voting (EPV) method is proposed to prioritize candidate driver genes. (D) demonstrates an example of TGBWDriver’s driver gene prediction performance on the BRCA cancer dataset, validating the model’s effectiveness in real-world applications.

### 4.3. Gene Representation Learning

#### 4.3.1. Gene Representation Learning Based on a Two-Layer GraphSAGE Architecture

GraphSAGE [55] is a graph neural network based on inductive learning. Its core idea is to generate embeddings for target nodes by sampling and aggregating the features of neighboring nodes. The key innovation of GraphSAGE lies in the fact that it does not learn fixed node embeddings but rather a general aggregation function. Unlike traditional Graph Convolutional Network (GCN) methods, GraphSAGE samples a fixed number of nodes within each node’s neighborhood and performs aggregation operations on these nodes, thereby enabling information propagation and fusion. This method can capture the graph’s topological structure and the complex relationships among nodes while maintaining computational efficiency.

In this study, TGBWDriver employed a two-layer GraphSAGE to learn the feature representations of gene and sample nodes. This multi-level information propagation mechanism enables the representation of nodes to rely not only on their directly adjacent nodes but also to incorporate broader graph structural information. The GraphSAGE model’s input consists of two parts: the known driver gene–sample association matrix and node attributes. Specifically, for each gene and sample node, TGBWDriver aggregated the features of its neighboring nodes via the adjacency matrix A using the GraphSAGE model and fused them with the node’s own features. We input the gene feature matrix $X_{g}$ and the sample feature matrix $X_{s}$ into GraphSAGE as the initial attributes of gene nodes and sample nodes, respectively. For convenience, we denote $H_{g}^{\left(0\right)}\;={\;X}_{g}\in R^{G\times N},\;H_{s}^{\left(0\right)}\;={\;X}_{s}\in R^{N\times G}$. For gene nodes, we aggregate neighbor information from sample nodes and combine it with the features of the gene nodes themselves to obtain updated gene features:

(2) $$H_{g}^{\left(l+1\right)}\;=\;\sigma \left(H_{g}^{\left(l\right)}W_{g,\mathrm{self}}^{\left(l\right)}+{\tilde{A}}_{\mathrm{gs}}H_{s}^{\left(l\right)}W_{g,\mathrm{neigh}}^{\left(l\right)}\right)$$

Here, $H_{g}^{\left(l\right)}$ represents the embedding of gene node g at the l-th layer, and ${\tilde{A}}_{\mathrm{gs}}$ is the normalized gene-to-sample association matrix computed as ${\tilde{A}}_{\mathrm{gs}}\;=\;D_{g}^{-1}A$ with $D_{g}(i,i)\;=\;\sum_{j=1}^{N}A_{\mathrm{ij}}$. $W_{g,\mathrm{self}}^{\left(l\right)}$ and $W_{g,\mathrm{neigh}}^{\left(l\right)}$ are the weight matrices at the l-th layer, and σ denotes the ReLU activation function.

Similarly, for sample nodes, the updated sample features are obtained by aggregating neighbor information from gene nodes and combining it with the node’s own features:

(3) $$H_{s}^{\left(l+1\right)}\;=\;\sigma \left(H_{s}^{\left(l\right)}W_{s,\mathrm{self}}^{\left(l\right)}+{\tilde{A}}_{\mathrm{sg}}H_{g}^{\left(l\right)}W_{s,\mathrm{neigh}}^{\left(l\right)}\right)$$

Here, $H_{s}^{\left(l\right)}$ represents the embedding of sample node s at the *l*-th layer, and ${\tilde{A}}_{\mathrm{sg}}$ is the normalized sample-to-gene association matrix computed as ${\tilde{A}}_{\mathrm{sg}}\;={\;D}_{s}^{-1}A^{T}$ with $D_{s}(j,j)\;=\;\sum_{i=1}^{G}A_{\mathrm{ij}}$. $W_{s,\mathrm{self}}^{\left(l\right)}$ and $W_{s,\mathrm{neigh}}^{\left(l\right)}$ are the weight matrices at the *l*-th layer, and σ denotes the ReLU activation function. After passing through the two-layer GraphSAGE architecture, the model obtains the representations of gene nodes and sample nodes, denoted as $H_{g}^{\left(2\right)}\in R^{G\times d}$ and $H_{s}^{\left(2\right)}\in R^{N\times d}$, respectively.

#### 4.3.2. Gene Representation Learning Based on Bidirectional Weighted Feature Aggregation Mechanism

To more precisely capture the bidirectional dependency between genes and samples, we propose, for the first time, a mechanism named “Bidirectional Weighted Feature Aggregation (BWFA)”. The information flow between genes and samples is not unidirectional but mutually dependent. Specifically, when each gene node aggregates features from sample nodes, it can dynamically adjust the strength of its relationships with different sample nodes based on the contextual information of the samples. As a result, samples with higher relevance will exert a greater influence on the feature representation of the gene node, while samples with weaker relationships to the gene will be assigned lower weights. This mechanism can capture the complex and asymmetric relationships between genes and samples, dynamically adjusting their mutual influences. Consequently, the node representations can more accurately reflect their roles and interrelationships within the graph. Specifically, let the updated gene and sample representations after two-layer GraphSAGE be denoted as $H_{g}^{\left(2\right)}\in R^{G\times d}$ and $H_{s}^{\left(2\right)}\in R^{N\times d}$, respectively. For simplicity, in the BWFA mechanism module, we set $G\;={\;H}_{g}^{\left(2\right)}$ and $S\;=\;H_{s}^{\left(2\right)}$. We first calculate the weighted aggregation from genes to samples.

First, the gene representations G and sample representations S learned by the two-layer GraphSAGE are mapped into a common space through learnable linear transformations:

(4) $$Q_{G}\;=\;GW_{Q},\;K_{S}\;=\;SW_{K},\;V_{S}\;=\;SW_{V}$$

Here, $Q_{G}$ represents the query vectors for samples, $K_{S}$ represents the key vectors for genes, and $V_{S}$ represents the value vectors for genes. $W_{Q}$,$W_{K}$ and $W_{V}$ are their corresponding transformation weight matrices. Then, the association score is calculated. Specifically, the association score is obtained by computing the dot-product similarity between the transformed embedding vectors and then applying Softmax normalization, rather than being a static value based on raw gene features or network topology. The association score matrix is defined as follows:

(5) $$E_{G‐S}\;=\;\mathrm{Softmax}(\frac{Q_{G}K_{S}^{T}}{\sqrt{d_{k}}})$$

Next, by taking a weighted sum of the sample features VS, we obtain the updated gene representation:

(6) $$G\prime \;={\;E}_{G‐S}V_{S}$$

Finally, the updated gene representation is obtained through a nonlinear transformation:

(7) $$\hat{G}\;=\;\phi (G\prime W_{\mathrm{pg}}+b_{\mathrm{pg}})$$

Here, ϕ(⋅) represents the ReLU activation function, $W_{\mathrm{pg}}\in R^{d_{k}\times d}$ is the projection matrix, and $b_{\mathrm{pg}}$ is the bias term. Next, we perform a similar operation to compute the weighted aggregation of samples with respect to genes:

(8) $$Q_{S}\;=\;SW_{Q},\;K_{G}\;=\;GW_{K},\;V_{G}\;=\;GW_{V}$$

(9) $$E_{S‐G}=\mathrm{Softmax}(\frac{Q_{S}K_{G}^{T}}{\sqrt{d_{k}}})$$

(10) $$S\prime ={\;E}_{S‐G}V_{G}$$

(11) $$\hat{S}=\phi (S\prime W_{\mathrm{ps}}+b_{\mathrm{ps}})$$

### 4.4. Driver Gene Prediction

We obtained the final gene feature representation $\hat{G}$ and sample feature representation $\hat{S}$, which are used for further similarity computation. First, we transform the dimensions of gene and sample features using Equations (13) and (14):

(12) $$H_{g}\;=\;\hat{G}\cdot W_{0}$$

(13) $$H_{s}=\hat{S}\cdot W_{1}$$

Here, $W_{0}\in R^{l\times k}$ and $W_{1}\in R^{l\times k}$ are the weight matrices of two linear layers, and $H_{g}\in R^{G\times k}$ and $H_{s}\in R^{N\times k}$ are the linearly transformed gene and sample features. Next, we adopt cosine similarity as the similarity metric to compute the relationship between genes and samples:

(14) $${\hat{A}}_{\mathrm{ij}}\;=\;\frac{1}{2}\left(\frac{h_{g_{i}}\cdot h_{s_{j}}^{T}}{\left\|h_{g_{i}}\left\|\cdot \right\|h_{s_{j}}\right\|}+1\right)$$

Here, $h_{g_{i}}\in H_{g}$ and $h_{\mathrm{sj}}\in H_{s}$ denote the feature representations of gene i and sample j, respectively, and ${\hat{A}}_{\mathrm{ij}}\in$ [0, 1] represents the probability that gene i is a driver gene in sample j.

In the optimization process of deep learning models, the choice of loss function is a critical factor that directly affects both the convergence speed and the final prediction accuracy. To address the limitations of standard binary cross-entropy loss in scenarios with extreme imbalance between positive and negative samples, this paper adopts a weighted binary cross-entropy loss function. This loss function introduces a balancing hyperparameter β to amplify the loss contribution of positive samples, thereby forcing the model to prioritize the minority class. It is defined as follows:

(15) $$L\;=\;-\frac{1}{\left|M\right|}\sum_{(i,j)\in M}\left[\beta \cdot A_{ij}\cdot \log({\hat{A}}_{ij})+(1-A_{ij})\cdot \log(1-{\hat{A}}_{ij})\right]$$

Here, M is the set of gene–sample pairs covered by the training mask (test samples are excluded in the K-fold cross-validation), |M| is the total number of elements in M, β is the hyperparameter weight assigned to positive samples, $A_{\mathrm{ij}}\in${0,1} is the ground-truth label, and ${\hat{A}}_{\mathrm{ij}}$ is the predicted probability of the positive class.

For each sample, all genes are sorted according to their predicted similarity scores in the corresponding column of the similarity matrix ${\hat{A}}_{\mathrm{ij}}$. To improve computational efficiency and focus on high-confidence candidates, only genes with somatic mutations in that sample are retained, ranked in descending order of their predicted scores, and the top 50% are selected to form the sample-specific candidate driver gene subset. To more effectively integrate gene ranking results across different samples at the cancer-specific level, we propose an Exponential Pairwise Voting (EPV) strategy for prioritizing candidate driver genes. In personalized driver gene analysis, gene rankings often vary substantially across samples, and information from higher-ranked positions is generally more discriminative than that from lower-ranked positions. The core idea of EPV is to perform pairwise comparisons of genes based on sample-level ranking lists and to introduce an exponential mechanism to modulate the contribution of ranking positions, such that comparisons involving higher-ranked genes contribute more to the overall score, while information from lower-ranked positions is retained with gradually diminishing influence. In this way, EPV effectively exploits sample heterogeneity while avoiding information loss caused by simple averaging or hard truncation strategies.

Suppose there are N samples. For each sample Rs (s = 1, 2, …, N), weights are assigned to the candidate driver genes according to their ranking positions, where genes with higher rankings receive larger weights. Let the rank of gene g in sample $R_{s}$ be $r_{s}\left(g\right)$ (counting from 1, where 1 indicates the highest rank). Then its weight is defined as:

(16) $$w_{s}(g)\;=\;e^{-\alpha \cdot (r_{s}\left(g)-1\right)}$$

Here, α is a hyperparameter that controls the exponential decay rate of the contribution of ranking positions to the voting process. This definition ensures that genes with higher rankings contribute more in pairwise comparisons, while the information from lower-ranked genes is still preserved but with gradually diminishing influence. If $g_{i}$ appears in sample $R_{s}$ and satisfies either condition (1), where $g_{j}$ also appears in sample $R_{s}$ and $g_{i}$ has a higher rank than $g_{j}$, or condition (2), where $g_{j}$ does not appear in sample $R_{s}$, then $g_{i}$ wins against $g_{j}$, and the voting strength is $w_{s}(g_{i})$; otherwise, the voting strength is 0:

(17) $$v_{s}(g_{i},g_{j})\;=\;\left\{\begin{array}{ll} w_{s}(g_{i}), & \mathrm{if}\;g_{i}\in R_{s}\;\mathrm{and}\;r_{s}(g_{i})<r_{s}(g_{j})\\ w_{s}(g_{i}), & \mathrm{if}\;g_{i}\in R_{s}\;\mathrm{and}\;g_{j}\notin R_{s}\\ 0, & \mathrm{otherwise} \end{array}\right.$$

For each gene $g_{i}$, its win strength is defined as the sum of voting strengths of this gene defeating other genes across all samples, while its loss strength is defined as the sum of voting strengths of other genes defeating this gene across all samples, as given by Equations (18) and (19), respectively:

(18) $$\mathrm{win}(g_{i})\;=\;\sum_{s=1}^{N}\sum_{j\neq i}v_{s}(g_{i},g_{j})$$

(19) $$\mathrm{loss}(g_{i})=\sum_{s=1}^{N}\sum_{j\neq i}v_{s}(g_{j},g_{i})$$

The final EPV score of gene $g_{i}$ is defined as the ratio of its win strength to the total comparative strength, i.e., the quotient of the win strength divided by the sum of the win strength and loss strength. A higher score indicates a stronger relative advantage of the gene in pairwise comparisons, making it more likely to be a driver gene. Finally, candidate genes are sorted in descending order according to their EPV scores to determine their priority. This score is given by Equation (20):

(20) $$P(g_{i})\;=\;\frac{\mathrm{win}(g_{i})}{\mathrm{win}(g_{i})\;+\;\mathrm{loss}(g_{i})}$$

## Figures and Tables

**Table 1:** Ablation study results of different components in TGBWDriver.

	One-Layer GraphSAGE	Two-Layer GraphSAGE	BWFA	EPV	Standard Condorcet Ranking	BRCA	LUAD	PRAD
		√	√	√		**0.469**	**0.481**	**0.144**
	√				√	0.383	0.421	0.097
	√			√		0.424	0.450	0.122
F1-score		√		√		0.463	0.472	0.134
	√		√	√		0.422	0.475	0.061
	√		√		√	0.275	0.330	0.083
		√			√	0.233	0.220	0.097
		√	√		√	0.300	0.330	0.122

Note: *√* indicates that the corresponding component or strategy is enabled in the experimental configuration.

**Table 2:** Sensitivity analysis of the weight parameter α in the Exponential Pairwise Voting strategy.

α	BRCA	LUAD	PRAD
0	0.300	0.330	0.280
0.1	0.427	0.469	0.357
0.2	0.452	0.480	0.382
0.3	0.463	**0.481**	0.382
0.4	0.463	0.480	0.382
0.5	0.463	0.478	0.382
0.6	0.466	0.472	**0.395**
0.7	0.466	0.472	0.382
0.8	0.466	0.472	0.382
0.9	**0.469**	0.472	0.382
1	0.466	0.472	0.382

**Table 3:** Number of genes (nodes) and interactions (edges) in the four networks.

	CPDB	STRING	PCNET	MULTINET
Nodes	13,627	13,179	19,781	14,398
Edges	504,378	336,548	2,424,724	109,567

## Data Availability

The source code and datasets used in this research can be downloaded from https://github.com/SCSMDyeah/TGBW.

## Supplementary Materials

The following supporting information can be downloaded at: https://www.mdpi.com/article/10.3390/ijms27094135/s1.

## Abbreviations

The following abbreviations are used in this manuscript:

BWFA	Bidirectional Weighted Feature Aggregation
EPV	Exponential Pairwise Voting
