# Supplementary material for: TGBWDriver: A Cancer Driver Gene Identification Method Based on GraphSAGE and Bidirectional Weighted Feature Aggregation
Source: Int J Mol Sci. 2026 May 5;27(9):4135. doi: 10.3390/ijms27094135 (PMC13163706; doi:10.3390/ijms27094135)
Supplement: Supplementary file 1 [file ijms-27-04135-s001.zip › ijms-4286916-supplementary.pdf]

Supplementary file of "TGBWDriver: A Cancer Driver Gene Identification Method Based on GraphSAGE and Bidirectional Weighted Feature Aggregation"

Supplementary Tables

Table S1. Methodological comparison of existing driver gene identification methods.

Algorithm type	Essential input data type(s)	Rationale	Example applications
Mutation frequency–based	mutation	Comparing observed mutation burden to expected background rates.	MutSigCV[**Error! Reference source not found.**], MuSiC[**Error! Reference source not found.**], and OncodriveFM[**Error! Reference source not found.**]
Network-based	PPI network, mutation , expression	Mutations in cancer driver genes tend to have widespread effects across molecular interaction networks.	HotNet2[7], DawnRank[8], PRODIGY[9]
Machine learning	Multi-omics data	The hidden patterns within the complex and highdimensional cancer data are difficult to analyse through statistical methods.	20/20+[13], DriverML[14], DORGE[15], IMCDriver
GNN	Multi-omics data	The topology of graphs, including the direct and indirect relationships between nodes (genes), can be learned	EMOGI[17], MTGCN[18], NIGCNDriver[20]

Table S2. Top 100 genes predicted by TGBWDriver in BRCA across four PPI networks

CPDB	STRING	PCNET	MULTINET
TP53	TP53	TP53	PIK3CA
PIK3CA	PIK3CA	PIK3CA	TP53
GATA3	GATA3	GATA3	GATA3
CDH1	CDH1	CDH1	CDH1
KMT2C	MAP3K1	KMT2C	RYR2
USH2A	KMT2C	PTEN	MAP3K1
MAP3K1	USH2A	NCOR1	RUNX1
NCOR1	NCOR1	USH2A	TBX3
NF1	RYR2	MAP3K1	NCOR1
NOTCH2	RUNX1	ARID1A	ARID1A
PTEN	ARID1A	RUNX1	PTEN
CHD8	SYNE1	SYNE1	SYNE1
SPEN	DNAH11	SPEN	PTPRD
RUNX1	TBX3	NF1	CHD8
DNAH11	PCLO	TBX3	HRNR
PTPRD	PTEN	PTPRD	PCLO
RYR2	AKT1	PIK3R1	NF1
TBX3	NF1	DNAH11	SPEN
SYNE1	PTPRD	AKT1	MAP2K4
RB1	MED12	SETD2	AKT1
PCLO	PIK3R1	CHD8	PIK3R1
AKT1	ARHGAP35	ARHGAP35	AFF2
ARID1A	SPEN	MED23	ATM
ATM	STAG2	BRCA1	GRIN2A
PIK3R1	GNAS	MED12	MAP3K13
TNFAIP3	ATM	RYR2	RET
ARHGAP35	HRNR	SF3B1	RB1
MAP2K4	ATR	ATM	MED23
MAP3K13	KMT2D	GNAS	ASXL1
MED12	TNFAIP3	CREBBP	BRCA1
KMT2D	FOXA1	KMT2D	ZNF217
HRNR	ZFP36L1	KMT2A	BRIP1
SF3B1	ACVR1B	ARID1B	GNAS
CUX1	SF3B1	RB1	FOXA1
ZFP36L1	NOTCH2	PCLO	SF3B1
ESR1	MED23	ERBB2	NOTCH2
ACVR1B	CHD8	KDM6A	TTN
ATRX	EPHA7	STAG2	FBXW7
FLNA	ATAD2	ZNF217	ACVR1B
STAG2	KMT2A	USP9X	TNFAIP3
GNAS	ARID1B	NOTCH2	IRS4
BRCA1	MAP3K13	ASXL1	MED12
KMT2A	RB1	ATR	ERBB4

BRIP1	ZNF217	MAP2K4	CTCF
ZNF217	ERBB2	FOXP1	ROS1
ERBB4	EXT2	ZFP36L1	FGFR2
FOXA1	EP300	XBP1	STAG2
ERBB2	USP9X	CHD4	ALK
FOXP1	SETD2	TNFAIP3	CUX1
MET	BRCA1	BRIP1	EXT2
USP9X	APC	ERBB3	GPR162
ARID2	ARID2	ATRX	USP9X
ATR	NOTCH1	ALK	ARID2
ERBB3	IRS4	EPHA7	ERBB2
ARID1B	ESR1	EPHB1	EP300
CASZ1	FBXW7	ACVR1B	ZFP36L1
FBXW7	CREBBP	ROS1	ATR
KDM6A	MAP2K4	RYR1	HIST1H2BC
AFF2	MUC16	FOXA1	FLNA
MED23	CASZ1	DMBT1	APC
APC	ASXL1	EXOC2	EGFR
IRS4	KRAS	MYH6	XBP1
CTCF	NCOA3	ATAD2	ATRX
ETV6	ALK	FLNA	KDM6A
CHEK2	BRIP1	GRIN2A	NOTCH1
FGFR2	DNMT3A	KCNN3	PRDM1
ASXL1	CTCF	ACTG1	ACTG1
NCOA3	FGFR2	CASZ1	MARK1
CREBBP	ACTG1	ETV6	RPGR
EPHB1	ATRX	CTCF	HIST1H3B
CASP8	EGFR	ZAN	CREBBP
RET	BCOR	ZMYM3	FOXP1
EGFR	RET	RET	MYB
EPHA7	FLNA	ESR1	SETD2
XBP1	PBRM1	NOTCH1	NCOA3
DICER1	ZMYM3	KIT	RYR1
NOTCH1	CHEK2	AFF2	PALB2
ZMYM3	EPHB1	BAP1	RNF8
GRIN2A	NOTCH3	MYB	MEN1
SETD2	ERBB4	BRCA2	PDGFRA
CNOT3	FLG	HRNR	BAZ2B
ROS1	ERBB3	FBXW7	ETV6
FOXO3	CDKN1B	GRM3	EPHA7
HIST1H3B	MYB	CUX1	BCOR
MYB	XBP1	ARID2	ARID1B
ATAD2	TTN	IRS4	ESR1
EP300	AFF2	EP300	CCNE1

PRDM1	MEN1	SPOP	CASZ1
GPS2	KDM6A	PIWIL1	MET
CCNE1	ETV6	ZNF532	SPOP
TBL1XR1	FOXP1	NUP210L	ATAD2
ZNF28	CNOT3	PBRM1	FLG
ACTG1	BRCA2	EGFR	AKT3
DDR2	DICER1	NCOA3	CHEK2
SPOP	MSH2	BRAF	ERBB3
KCNN3	BAP1	SCN2A	FGFR1
CBL	MET	CBLB	CNOT3
PDGFRA	GRIN2A	PRDM1	TG
EXOC2	PDGFRA	TTN	WT1
PALB2	MUC4	PDGFRA	KCNN3

Table S3. Top 100 genes predicted by TGBWDriver in LUAD across four PPI networks

CPDB	STRING	PCNET	MULTINET
KRAS	TP53	TP53	TP53
TP53	MUC16	LRP1B	KRAS
MUC16	KRAS	ZFHX4	PTPRD
ZFHX4	PTPRD	MUC16	FAT4
NAV3	STK11	STK11	KEAP1
LRP1B	NLRP3	PKHD1	LRRC7
STK11	COL11A1	KRAS	STK11
COL11A1	TLR4	COL11A1	EGFR
PTPRD	KEAP1	ZNF536	NLRP3
ROBO2	ASPM	CDH10	EPHA3
FAT4	RELN	CNTNAP2	CNTNAP2
LRRC7	VCAN	KEAP1	PRDM9
PKHD1	LRRC7	NAV3	RIMS2
KEAP1	FAT4	LRRC7	CDH10
EGFR	RIMS2	COL12A1	TLR4
ZNF536	HCN1	TLR4	COL12A1
CNTNAP2	EGFR	PTPRD	GRM1
TLR4	NF1	EGFR	VCAN
ZNF479	PRDM9	ROBO2	MUC5B
EYS	CDH10	FAT1	PIK3CG
SMARCA4	RUNX1T1	VCAN	RELN
HCN1	COL12A1	EYS	ZNF521
ASPM	ATM	MGA	SLIT2
KMT2C	PIK3CG	FAT4	ATM
CACNA2D1	ERBB4	EPHA5	ZEB2
NLRP3	KMT2D	RELN	KDR
CDH10	GRIK3	ZNF479	NF1
ATM	EPHA3	SLIT2	NAV3

NOTCH1	CNTNAP2	NLRP3	ASPM
EPHA5	ALMS1	PRDM9	OBSCN
RELN	SMARCA4	ATRX	EPHA5
ERBB4	ZNF479	GLI3	TRO
EPHA3	MGA	GRM1	FAT1
VCAN	MKI67	CCKBR	FANCM
GRIK3	ROBO2	PTPRU	ZFHX3
ALMS1	KMT2C	RIMS2	CTNNB1
ZNF521	INHBA	KMT2C	PEG3
ARID1A	CACNA2D1	MKI67	CDH11
COL12A1	BRAF	EPHA3	ADCY1
MGA	CTNNB1	CACNA2D1	COL4A2
ABCB5	ADCY1	DNAH3	SMARCA4
NF1	GLI3	NF1	RYR2
RIMS2	ARID1A	NOTCH1	MKRN3
GRM1	RBM10	ABCB5	PIK3C2B
UBA6	NTRK3	ARID1A	NTRK3
FAT1	DNAH3	ADCY1	ARID2
CTNNB1	FANCM	ERBB4	INHBA
SLIT2	COL4A2	ZNF521	NOTCH1
MKI67	SLIT2	ZFHX3	ERBB4
DNAH3	RB1	INHBA	BRCA1
PTPRU	CDH11	FANCM	BRAF
RUNX1T1	UBA6	DACH1	HCN1
PRDM9	NOTCH1	BRAF	HMCN1
TKTL2	DACH1	PIK3CA	PIK3CA
PIK3C2B	CD1B	CTNNB1	ATRX
CCKBR	ZFHX3	ATM	MKI67
FANCM	ATRX	KDR	GLI3
BRAF	PIK3C2B	CREBBP	WRN
RB1	KDR	COL4A2	RUNX1T1
NRG3	SLC6A2	KMT2D	NELL1
PDGFRA	GRM1	SMARCA4	SPTA1
PIK3CG	BRCA1	PIK3C2B	ROBO2
MET	EPHA5	ASPM	RGS7
ZEB2	FAT1	ZEB2	RBM10
COL4A2	ZEB2	RB1	SATB2
TRHDE	DNER	HCN1	RB1
BRCA1	NRG3	PPP1R3A	LGR5
ATRX	TBX22	KIF21A	PPP1R3A
RBM10	CDKN2A	GRM8	JAK2
MKRN3	PDGFRA	NTRK3	SLC5A7
SATB2	ZNF521	UBA6	MGA
DIP2C	P2RY8	ALMS1	CDKN2A

INHBA	SATB2	CTNNA3	CDYL
ZFHX3	GRM8	CDH11	DNER
SETD2	MET	CD1B	MET
SPHKAP	APC	SETD2	LAMA1
TFAP2D	FOLH1	PDGFRA	STIM1
ROS1	PIK3CA	ARHGEF12	ARID1A
EPB41L3	NFE2L2	SLC6A2	PASK
GRM8	ERBB2	EPRS	RASA1
CDH11	TFAP2D	GRIK3	FLNC
KDM6A	FLT3	RBM10	TKTL2
KMT2D	SMAD4	SATB2	TIGD4
RGS7	XRN1	RUNX1T1	RAF1
XRN1	PTPRU	PIK3CG	KIF21A
KDR	ARHGAP35	JAK2	GNAS
FBXW7	PPP1R3A	ERBB2	MARK1
APC	KIF21A	BRCA1	CRB1
JAK2	ARID1B	STIM1	VEGFC
PIK3CA	CCKBR	TKTL2	FBXW7
ARHGAP35	CTNNA3	P2RY8	CENPF
DACH1	RGS7	NRG3	XRCC5
FLT3	SETD2	FLT3	ITK
CDKN2A	GNAS	HEPACAM2	UBA6
PTCH1	FBXW7	ROS1	LATS2
BAZ2B	NTRK1	EP300	SETD2
P2RY8	ARHGEF12	MET	PDGFRA
NTRK3	FPR1	MKRN3	NRG3
PDYN	TKTL2	TRHDE	SI
CTNNA3	SPHKAP	ARID1B	KHSRP

Table S4. Top 100 genes predicted by TGBWDriver in PRAD across four PPI networks

CPDB	STRING	PCNET	MULTINET
TP53	TP53	TP53	TP53
SPOP	SPOP	SPOP	SPOP
FOXA1	FOXA1	FOXA1	FOXA1
KMT2D	KMT2D	KDM6A	TTN
ATM	ATM	KMT2D	KDM6A
KDM6A	KDM6A	ZFHX3	ATM
PTEN	PTEN	KMT2C	SYNE1
ZFHX3	TTN	CTNNB1	PTEN

KMT2C	ZFHX3	TTN	ZFHX3
CTNNB1	SPTA1	ATM	CTNNB1
ZMYM3	ZMYM3	BRAF	OBSCN
SPTA1	KMT2C	ZMYM3	ZMYM3
TTN	BRAF	PTEN	BRAF
BRAF	PIK3CA	CDKN1B	PIK3CA
RYR2	CTNNB1	HMCN1	CDKN1B
PIK3CA	PTPRC	LRP1B	APC
CDKN1B	MYH8	BRCA2	ANK2
APC	APC	AKT1	SPTA1
AKT1	SYNE1	ARID1A	MYH10
NKX3-1	TMPRSS2	FBN3	FAT3
ARID1A	PIKFYVE	PIK3CG	AKAP9
TRIOBP	MUC16	APC	PIK3CG
TMPRSS2	BRCA2	MUC5B	BRCA2
AKAP9	ZBTB16	AKAP9	ZBTB16
BRCA2	AKT1	TMPRSS2	AKT1
SLC6A5	NKX3-1	KIF1A	NKX3-1
MUC16	COL11A1	CACNA1E	ARID1A
ABCA4	CDKN1B	PIK3CA	RYR2
SCN10A	TRPA1	BSN	CACNA1E
ZBTB16	AKAP9	SLITRK5	SRCAP
RYR3	MYH15	CHD1	GRIN2B
PTPRC	ARID1A	ZBTB16	IDH1
IDH1	OBSCN	MED12	MGA
ITGAD	LCE1C	EDF1	CSF1R
RGS6	FRAS1	FEZF1	ESF1
MED12	IDH1	NKX3-1	COL12A1
LRP2	PIK3CG	BICD1	TRPA1
GSTO1	TNXB	MYO6	ACVRL1
EXOSC10	FLG	RYR1	MTOR
DMD	TIAM2	COL12A1	CHD1

MYO6	NCOR2	RYR2	NCOR2
KIF1B	DSG4	SPEG	TPR
EFCAB6	KPNA6	DNAH3	MED12
ALPK3	CNTN4	SRCAP	RRS1
OBSCN	MED12	PTCH2	USE1
COL7A1	VWA8	MGA	TIE1
HCN1	MGA	PYHIN1	SLC34A2
LTBP4	MYH6	VWF	ADD2
TIAM2	CHD1L	DNAH9	PCDH17
CHD1	USH2A	NCOR2	EIF4G3
TFDP1	DSCAM	IDE	RYR1
KMT2A	LMTK2	NIPBL	TFDP1
ERF	MYH4	COL5A1	POM121
SALL1	PTPRD	UBR4	PDE4D
CHD4	POLR2B	MXRA5	SPEG
ARNTL2	KCNB2	RSBN1	MUC5B
COL1A2	EPB41L3	B3GALT4	CDK12
KCNJ5	CHD1	KRT27	SI
SETD2	PIK3R1	SUN2	KCNJ5
SCN4A	KRT27	MICALL1	PLCE1
TRIM33	CDK12	SFRP2	RIMS2
LCE2D	KCNA5	COL7A1	RXRG
TNXB	MYO7A	MED13	USP22
PCDHA7	GRM5	ANK2	ITPR2
POM121	MC3R	MUC16	RBL2
TPR	MYH11	POM121	MED13L
PDZRN3	HRAS	LRCH4	CNOT3
NCOR2	SETD2	LYST	EHHADH
NPC1	LRRC8B	PCDHB6	FAM83D
PRPF19	LRP6	FUK	SETD2
FARP2	KSR1	MAST2	BSN
AJAP1	FBN3	MSI2	MYCBP2

TBX5	POLQ	SLC6A5	FOXP1
PIK3CG	MUC5B	TMEM87A	CADPS
PADI4	CFH	CDH9	PIK3R1
NCKAP1	NOS2	LCE2D	PTPRK
SLC34A2	NOTCH3	IL3RA	AHRR
EIF4G3	COL5A1	CDH8	ERF
BRINP1	TFDP1	LTBP4	SLC4A1
MYCBP2	CACNA1E	ZNF99	ADCY9
ACVRL1	SPEN	LAMB1	WNK4
SUGP2	ARFGEF1	PIK3R1	PLCG2
MUC5B	BRINP1	GSTM5	CD48
FOXP1	LRCH4	TGFBR1	CHD1L
ABCA12	MTOR	LSR	EHBP1
ZSCAN4	PRPF19	FRAS1	GAD2
HOGA1	LILRA4	MARK4	MMP16
CTNNAL1	HSD17B3	TRIOBP	PASK
CDK12	MARK4	ZNF827	FUK
ITGA4	CARD16	EFCAB6	KIF21A
GFPT2	LCE2D	SCN4A	CTTNBP2
HNRNPR	ITGB4	SYNE2	IFT122
RANBP2	MED13L	CSMD2	FGB
CACNA1G	MTTP	USP6	SEL1L3
ATIC	SCN1A	SCNN1B	MED13
MYO7A	FBN1	RELN	KCNF1
HRAS	CSF1R	KMT2A	EIF2AK3
GOLGA3	EPB41L2	SALL1	LAMA1
ANK2	JMJD1C	XIRP1	CDH6
ZBED1	MAP1A	MED15	RUNX3

Novel driver gene predictions in LUAD supported by literature evidence

Taking lung adenocarcinoma (LUAD) as an example, we similarly identified multiple genes with high prediction scores that are not listed in the NCG database, with

independent literature supporting their critical roles in LUAD. Specifically, at the level of environmental exposure and carcinogenic mechanisms, studies have identified SYNE1[34] as a diagnostic/prognostic biomarker for assessing microplastic exposure in LUAD, revealing that microplastics can promote metal ion deposition and oncogenic signaling, thereby participating in the progression of LUAD. At the level of molecular pathological mechanisms, tissue microarray (TMA) analysis of human tumor tissues revealed that UBR4[35] expression levels are significantly correlated with LUAD progression and PINK1 levels (a key mediator of mitophagy-mediated mitochondrial quality control), uncovering the pathological mechanisms underlying UBR4-mediated LUAD development and highlighting the potential therapeutic value of targeting UBR4 as a viable treatment approach for lung cancer. In terms of driver genes and therapeutic targets, LUAD with ALK[36] rearrangements exhibits reduced tertiary lymphoid structures, which are closely associated with tumor progression and may contribute to poor response to immune checkpoint inhibitors; MYC/MAX[37]-transactivated LINC00958 promotes malignant behavior in LUAD by activating HOXA1 to induce oncogenic transcriptional reprogramming. Regarding diagnostic and prognostic biomarkers, bioinformatics analysis revealed that FANCI[38] is a core gene in LUAD, highly expressed in LUAD samples, and associated with metastasis and poor prognosis; integrative bioinformatics analysis combined with experimental validation demonstrated that kinesin family genes KIF11[39] (along with KIF4A and KIF20A) can serve as prognostic biomarkers for LUAD, with in vitro experiments confirming that silencing these genes effectively inhibits the proliferation, invasion, and migration of LUAD cells, suggesting that this biomarker combination has potential as both a biomarker and therapeutic target for LUAD patients. Together, the above literature evidence supports, from multiple levels including environmental exposure, molecular pathology, driver genes, and diagnostic prognosis, that these high-scoring genes predicted by TGBWDriver possess clear biological functions and clinical relevance, further validating the reliability of this method in identifying novel driver genes across different cancer types.

Novel driver gene predictions in LUAD supported by literature evidence

Taking prostate adenocarcinoma (PRAD) as an example, we similarly identified multiple genes with high prediction scores that are not listed in the NCG database, with independent literature supporting their critical roles in PRAD. Specifically, in terms of therapeutic targets, ORC1 is a previously unreported direct downstream target gene of AR in PRAD cells, where AR activates ORC1 expression to promote PRAD progression and enzalutamide resistance, potentially providing a new therapeutic target for PRAD treatment; GLIS1 has been identified as a potential prognostic biomarker for prostate cancer and a therapeutic target regulating anti-tumor immune responses. At the level of transcriptional regulation and epigenetics, partial correlation analysis confirmed that BAZ1A, together with VDR complex components identified by RIME, significantly enhances the correlation between VDR and target genes specifically in AA prostate cancer, indicating that VDR transcriptional control is strongest in AA prostate cells and is distorted through BAZ1A-dependent regulation of VDR function; gene set enrichment analysis showed that DNMT3A expression is closely associated with cell cycle G2/M

checkpoint regulation, suggesting its role in prostate cancer progression. Regarding diagnostic and prognostic biomarkers, systematic cross-comparison of epithelial-mesenchymal transition (EMT)-related genes, validated target genes of miR-143-3p, and negatively correlated target genes identified MYO6 as a potential candidate gene in prostate cancer; additionally, genes such as BUB1B can serve as biomarkers to assist in the diagnosis and prognosis of prostate cancer. Together, the above literature evidence supports, from multiple levels including therapeutic targets, transcriptional regulation, epigenetics, and diagnostic prognosis, that these high-scoring genes predicted by TGBWDriver possess clear biological functions and clinical relevance, further validating the reliability of this method in identifying novel driver genes across different cancer types.

Supplementary Figure

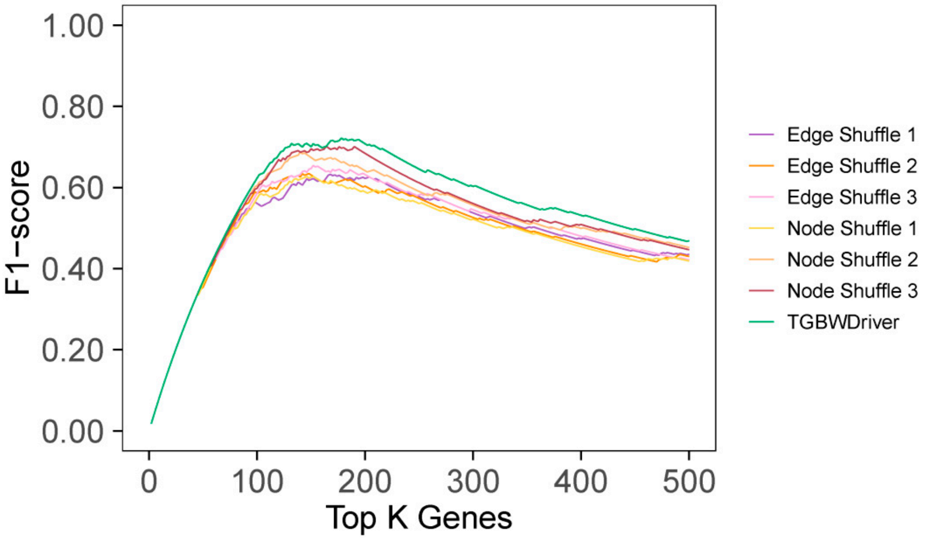

Figure S1. Negative control results on the BRCA dataset. Performance comparison of TGBWDriver on the original CPDB network versus six negative control networks, evaluated using F1-score.

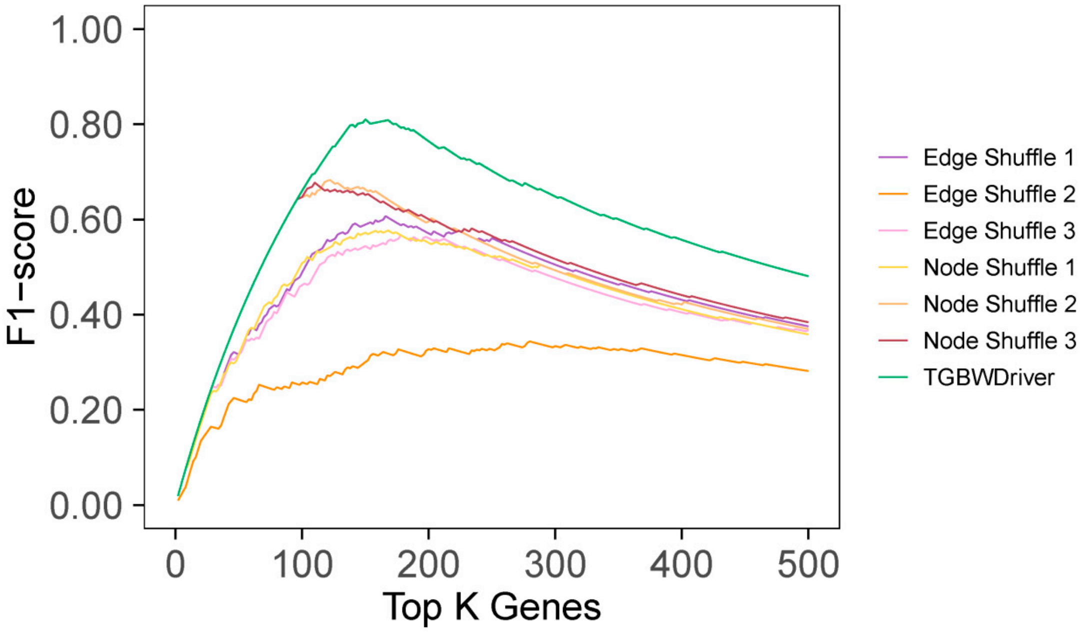

Figure S2. Negative control results on the LUAD dataset. Performance comparison of TGBWDriver on the original CPDB network versus six negative control networks, evaluated using F1-score.

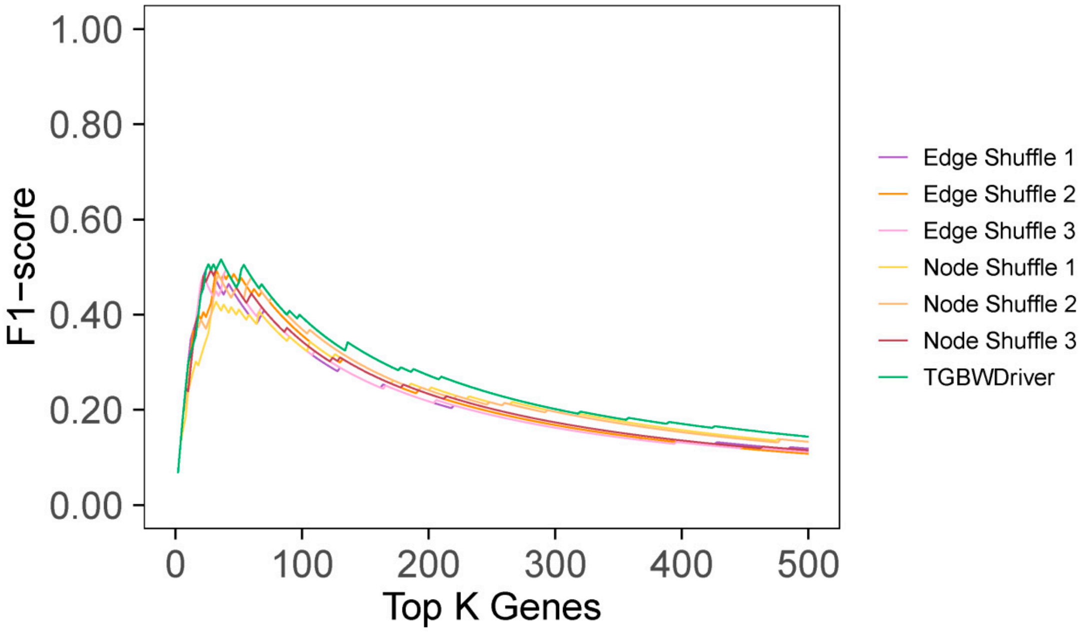

Figure S3. Negative control results on the PRAD dataset. Performance comparison of TGBWDriver on the original CPDB network versus six negative control networks, evaluated using F1-score.

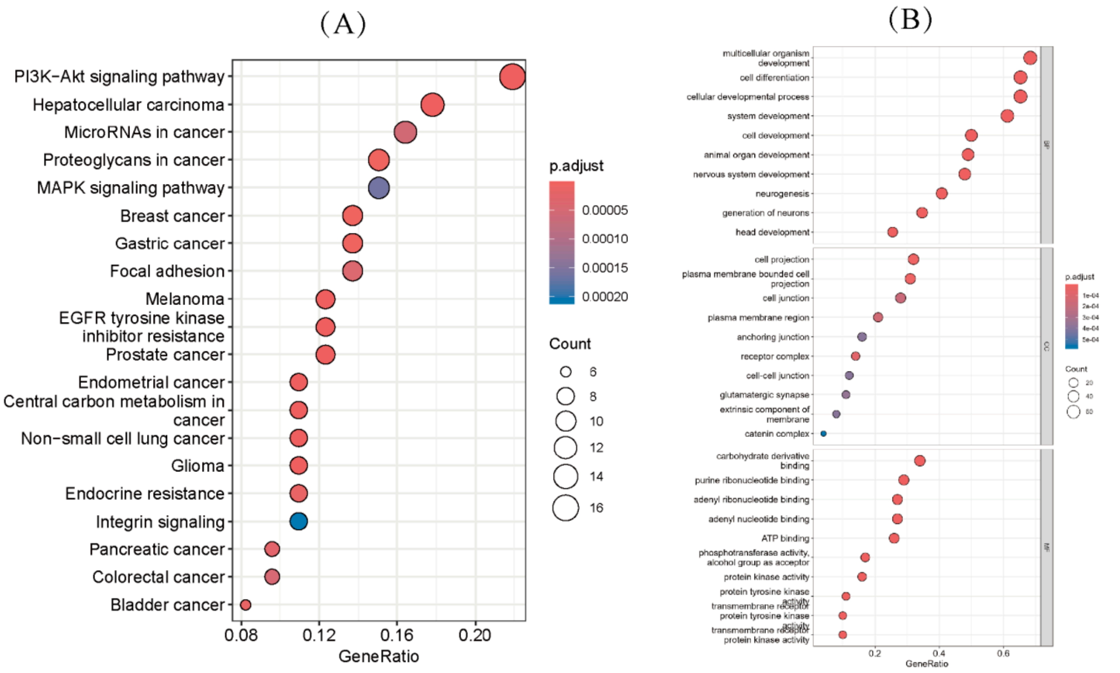

Figure S4. GO and KEGG enrichment analysis of genes predicted by TGBWDriver in LUAD. Functional enrichment analysis was performed on the top 100 genes predicted by TGBWDriver in the BRCA dataset using the R package clusterProfiler.(a) KEGG pathway enrichment results.(b) GO enrichment results for Biological Process (BP), Cellular Component (CC), and Molecular Function (MF), showing the top 10 most significant GO terms in each category.The size of each bubble represents the number of enriched genes (Count), and the color indicates the adjusted significance level (adjusted P value).

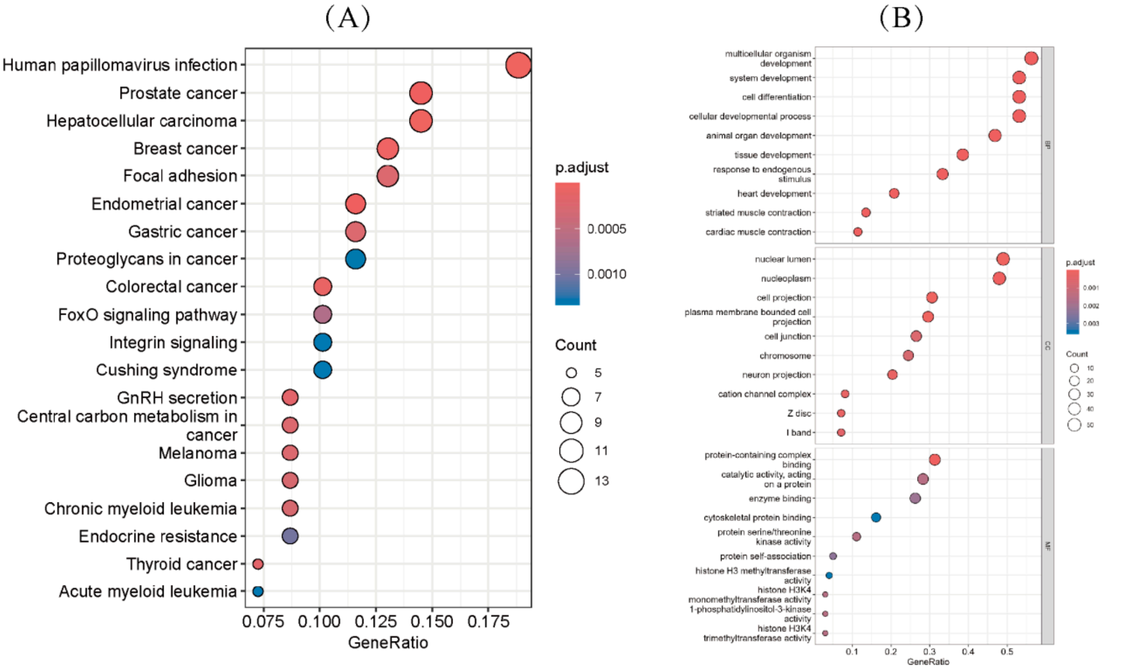

Figure S5. GO and KEGG enrichment analysis of genes predicted by TGBWDriver in

PRAD. Functional enrichment analysis was performed on the top 100 genes predicted by TGBWDriver in the BRCA dataset using the R package clusterProfiler.(a) KEGG pathway enrichment results.(b) GO enrichment results for Biological Process (BP), Cellular Component (CC), and Molecular Function (MF), showing the top 10 most significant GO terms in each category.The size of each bubble represents the number of enriched genes (Count), and the color indicates the adjusted significance level (adjusted P value).
